# Pulsed-field ablation for atrial fibrillation with an extended protocol: the extended PFA dose study

**DOI:** 10.1093/europace/euag253

**Published:** 2026-09-03

**Authors:** Corinne Isenegger, Alexander Breitenstein, Niklas Stauffer, Pascal Koepfli, David Siemer, Fabian Jordan, Sven Knecht, Philipp Krisai, Gian Völlmin, Leon Scharf, Jeanne du Fay de Lavallaz, Behnam Subin, Reto Stump, David Spreen, Felix Mahfoud, Christian Sticherling, Michael Kühne, Patrick Badertscher

**Affiliations:** Department of Cardiology, University Hospital Basel, University of Basel, Petersgraben 4, Basel 4031, Switzerland; Cardiovascular Research Institute Basel, University Hospital Basel, University of Basel, Petersgraben 4, Basel 4031, Switzerland; Department of Cardiology, University Hospital Zürich, Zürich, Switzerland; Department of Cardiology, Kantonsspital Baden, Baden, Switzerland; Department of Cardiology, Kantonsspital Baden, Baden, Switzerland; Department of Cardiology, Kantonsspital Baden, Baden, Switzerland; Department of Cardiology, University Hospital Zürich, Zürich, Switzerland; Department of Cardiology, University Hospital Basel, University of Basel, Petersgraben 4, Basel 4031, Switzerland; Cardiovascular Research Institute Basel, University Hospital Basel, University of Basel, Petersgraben 4, Basel 4031, Switzerland; Department of Cardiology, University Hospital Basel, University of Basel, Petersgraben 4, Basel 4031, Switzerland; Cardiovascular Research Institute Basel, University Hospital Basel, University of Basel, Petersgraben 4, Basel 4031, Switzerland; Department of Cardiology, University Hospital Basel, University of Basel, Petersgraben 4, Basel 4031, Switzerland; Cardiovascular Research Institute Basel, University Hospital Basel, University of Basel, Petersgraben 4, Basel 4031, Switzerland; Department of Cardiology, University Hospital Basel, University of Basel, Petersgraben 4, Basel 4031, Switzerland; Cardiovascular Research Institute Basel, University Hospital Basel, University of Basel, Petersgraben 4, Basel 4031, Switzerland; Department of Cardiology, University Hospital Basel, University of Basel, Petersgraben 4, Basel 4031, Switzerland; Cardiovascular Research Institute Basel, University Hospital Basel, University of Basel, Petersgraben 4, Basel 4031, Switzerland; Department of Cardiology, University Hospital Basel, University of Basel, Petersgraben 4, Basel 4031, Switzerland; Cardiovascular Research Institute Basel, University Hospital Basel, University of Basel, Petersgraben 4, Basel 4031, Switzerland; Department of Cardiology, University Hospital Basel, University of Basel, Petersgraben 4, Basel 4031, Switzerland; Cardiovascular Research Institute Basel, University Hospital Basel, University of Basel, Petersgraben 4, Basel 4031, Switzerland; Department of Cardiology, University Hospital Basel, University of Basel, Petersgraben 4, Basel 4031, Switzerland; Cardiovascular Research Institute Basel, University Hospital Basel, University of Basel, Petersgraben 4, Basel 4031, Switzerland; Department of Cardiology, University Hospital Basel, University of Basel, Petersgraben 4, Basel 4031, Switzerland; Cardiovascular Research Institute Basel, University Hospital Basel, University of Basel, Petersgraben 4, Basel 4031, Switzerland; Department of Cardiology, University Hospital Basel, University of Basel, Petersgraben 4, Basel 4031, Switzerland; Cardiovascular Research Institute Basel, University Hospital Basel, University of Basel, Petersgraben 4, Basel 4031, Switzerland; Department of Cardiology, University Hospital Basel, University of Basel, Petersgraben 4, Basel 4031, Switzerland; Cardiovascular Research Institute Basel, University Hospital Basel, University of Basel, Petersgraben 4, Basel 4031, Switzerland; Department of Cardiology, University Hospital Basel, University of Basel, Petersgraben 4, Basel 4031, Switzerland; Cardiovascular Research Institute Basel, University Hospital Basel, University of Basel, Petersgraben 4, Basel 4031, Switzerland; Department of Cardiology, University Hospital Basel, University of Basel, Petersgraben 4, Basel 4031, Switzerland; Cardiovascular Research Institute Basel, University Hospital Basel, University of Basel, Petersgraben 4, Basel 4031, Switzerland

**Keywords:** atrial fibrillation, Pulmonary vein isolation, Pulsed-field ablation

## Abstract

**Graphical Abstract:**
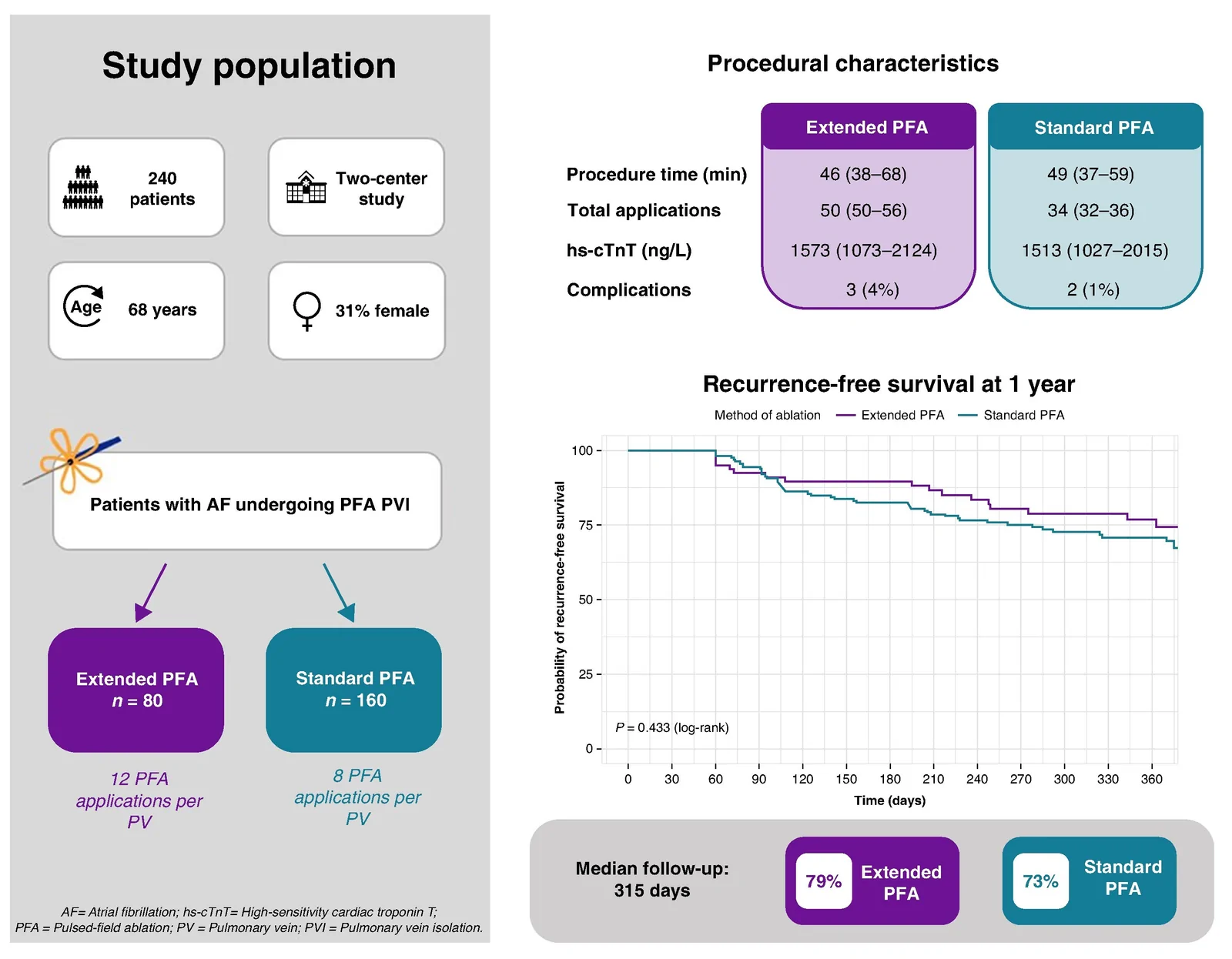

Pulsed-field ablation (PFA) has rapidly emerged as a preferred ablation modality for atrial fibrillation (AF) due to its favourable safety and efficiency profile.^1–3^ While clinical adoption of pentaspline PFA systems is widespread, data on modified application strategies beyond manufacturer-recommended protocols remain limited. Key procedural aspects, including the optimal number of applications per pulmonary vein (PV), remain poorly defined. Large registries have demonstrated safety and efficacy of PFA but inconsistently report the number of applications per PV, leaving an important procedural aspect insufficiently characterized.^2–4^ Whether increasing lesion ‘dose’ translates into improved clinical outcomes remains unknown.

This study aimed to compare an extended PFA dosing protocol (12 applications per PV) with a standard protocol (eight applications) regarding procedural characteristics, safety, and efficacy.

In this two-centre study, consecutive patients with paroxysmal or persistent AF undergoing index pulmonary vein isolation (PVI) using PFA between January 2022 and November 2024 were included. Patients with prior ablation or additional lesion sets were excluded. All patients provided written informed consent, and the study was approved by the local ethics committees.

Patients were treated using either a standard protocol (eight applications per PV)^5^ or an extended protocol (12 applications). In the extended protocol, four additional applications were delivered after the standard applications, with anterior tilt for two applications and posterior tilt for two applications to enhance circumferential lesion coverage. Procedures were performed using a pentaspline PFA catheter (FARAPULSE, Boston Scientific). Acute PVI was confirmed at the end of the procedure with assessment of entrance/exit block using a pacing protocol as previously described^6^ and/or electroanatomical mapping. The primary endpoint was freedom from atrial arrhythmia recurrence at 12 months after a 60-day blanking period, defined according to current guideline recommendations.^7^ Secondary endpoints included procedural characteristics and safety outcomes. Follow-up consisted of outpatient visits at 3, 6, and 12 months including ECG and Holter monitoring. Propensity score matching (1:2) was performed to adjust for baseline differences. Statistical analyses were conducted using standard methods.

After propensity score matching, 240 patients were included (extended: *n* = 80; standard: *n* = 160) with comparable baseline characteristics. Median age was 68 [60–74] years, 31% were female and 68% had paroxysmal AF. Procedural duration was similar between groups (46 [38–68] vs. 49 [37–59] min, *P* = 0.522), while fluoroscopy time was shorter in the extended protocol (9 [7–11] vs. 11 [8–14] min, *P* < 0.001). The number of applications was higher in the extended group (50 [50–56] vs. 34 [32–36], *P* < 0.001). Acute PVI was achieved in all patients. Post-procedural hs-cTnT levels were higher in the extended group (1789 [1330–2468] vs. 1513 [1027–2015] ng/L, *P* = 0.008). In a subset of patients, total antral scar area was larger with the extended protocol (12 [10–12] vs. 8 [7–9] cm^2^, *P* = 0.015). Complication rates were low and comparable between groups (4% vs. 1%, *P* = 0.337), including two cases of pericardial tamponade and one case of haemolysis in the extended group and one pericardial tamponade and one transient ST-segment elevation due to air embolism in the standard group. During a median follow-up of 315 [207–435] days, arrhythmia-free survival did not differ significantly between groups (79% vs. 73%, *P* = 0.433; *Graphical Abstract*). The distribution of recurrent arrhythmia types was similar between groups. Repeat ablation was performed in 12% of patients without significant differences between groups, and PV reconnection rates were similar.

Despite these markers of more extensive myocardial injury and larger lesion sets, clinical outcomes were unchanged. Arrhythmia-free survival at 12 months did not differ between groups (79% vs. 73%, *P* = 0.433). Repeat ablation rates and PV reconnection rates were also similar. Complication rates were low and comparable, although one case of haemolysis occurred in the extended group following a high number of applications.

These findings provide mechanistic and clinical evidence that increasing PFA ‘dose’ does not translate into improved efficacy. While additional applications resulted in greater biomarker release and larger scar areas, this did not improve rhythm outcomes, suggesting that lesion quantity alone is not the primary determinant of durable PVI.

Instead, our data support a paradigm in which effective lesion distribution and catheter–tissue interaction outweigh simple energy escalation.^8,9^ This observation is consistent with prior studies demonstrating comparable outcomes with simplified and standard PFA protocols.^10^ Taken together, both dose escalation and dose reduction strategies appear to converge on similar clinical efficacy, reinforcing that PFA effectiveness is not dose-driven within currently used ranges. Importantly, increasing application numbers may not be benign. The occurrence of haemolysis in the extended group, together with prior reports linking high cumulative energy delivery to haemolysis and renal injury, underscores the potential safety trade-off of excessive applications.^4^

This study is limited by its non-randomized design and moderate sample size, with potential residual confounding despite propensity matching. No systematic screening for haemolysis was performed in all patients. Residual confounding related to operator experience and learning curve effects cannot be fully excluded. Findings may not be generalizable to other PFA platforms.

In conclusion, escalating PFA application number increases myocardial injury and lesion size but does not improve clinical outcomes. These data challenge a dose-response paradigm for PFA and support a strategy focused on optimized lesion delivery rather than energy escalation.

## Data Availability

The datasets generated during and/or analysed during the current study are available from the corresponding author on reasonable request.
